# 晚期NSCLC患者EGFR-TKI治疗过程中血清多肽变化及其临床意义的探索性研究

**DOI:** 10.3779/j.issn.1009-3419.2016.09.08

**Published:** 2016-09-20

**Authors:** 子赫 王, 传昊 汤, 毅 刘, 彬 许, 海峰 秦, 阳阳 雷, 红军 高, 昆 何, 晓晴 刘

**Affiliations:** 1 100071 北京，军事医学科学院附属医院肺部肿瘤科 Department of Lung Cancer, Affiliated Hospital of Academy of Military Medical Sciences, Beijing 100071, China; 2 100071 北京，军事医学科学院附属医院肿瘤学研究室 Laboratory of Oncology, Affiliated Hospital of Academy of Military Medical Sciences, Beijing 100071, China; 3 100850 北京，国家生物医学分析中心 National Center of Biomedical Analysis, Beijing 100850, China

**Keywords:** 基质辅助激光解析电离飞行时间质谱, 肿瘤蛋白质组学, 表皮细胞生长因子受体, 酪氨酸激酶抑制剂, 肺肿瘤, Matrix-assisted laser desorption/ionization time-of-flight mass spectrometry, Cancer proteomics, Epidermal growth factor receptor, Tyrosine kinase inhibitor, Lung neoplasms

## Abstract

**背景与目的:**

本研究旨在应用基质辅助激光解析离子化-时间飞行质谱仪（matrix-assisted laser desorption ionization time-of-?ight mass spectrometry, MALDI-TOF-MS）检测晚期非小细胞肺癌（non-small cell lung cancer, NSCLC）患者在接受表皮生长因子受体酪氨酸激酶抑制剂（epidermal growth factorreceptor tyrosine kinase inhibitors, EGFR-TKIs）治疗过程中血清多肽的变化并探索其临床意义。

**方法:**

收集34例接受EGFR-TKI治疗的晚期NSCLC患者TKI治疗前、最佳疗效时及疾病进展后的自身配对血清样本102份。处理血清样本并应用MALDI-TOF-MS检测，得到质谱图后使用CPT统计软件进行分析，鉴定出差异多肽，并对其临床意义进行分析。

**结果:**

34例接受EGFR-TKI治疗的患者无完全缓解（complete response, CR）患者，部分缓解（partial response, PR）11例，疾病稳定（stable disease, SD）23例，中位无进展生存期（progression-free survival, PFS）为8.0个月（95%CI: 6.6-11.2）；中位总生存期（overall survival, OS）为11.4个月（95%CI: 10.6-16.5）。对TKI治疗的三个不同时间点的血清进行质谱检测，结果显示三个时间点多肽指纹图谱均不相同；配对分析最佳疗效时与基线时质谱数据经CPT软件共鉴定出差异多肽峰87个，筛选出两组间有统计学差异[*P* < 0.001、曲线下面积（area under curve, AUC）≥0.9]的多肽峰1个；疾病进展时与基线时共鉴定出差异多肽峰96个，筛选出两组间有统计学差异（*P* < 0.001, AUC≥0.9）的多肽峰3个；最佳疗效时与疾病进展时共鉴定出差异多肽峰115个，筛选出两组间有统计学差异（*P* < 0.001, AUC≥0.9）的多肽峰4个。

**结论:**

NSCLC患者TKI治疗过程中血清多肽存在动态变化，差异多肽可能与治疗效果、疾病进展相关，差异多肽的特性、临床意义需进一步鉴定和验证。

## Abstract (en)

**Background and objective:**

This study aimed at using matrix-assisted laser desorption ionization -time of flight mass spectrometer (matrix-assisted laser desorption ionization time-of-flight mass spectrometry, MALDI-TOF-MS) screening the difference serum peptides during epidermal growth factor tyrosine kinase inhibitors (EGFR-TKIs) treatment and exploring their significance of advanced NSCLC patients.

**Methods:**

Collect 102 serum samples from 34 advanced NSCLC patients, which are before TKI treatment, best effect of treatment and after progession. Peptides were extracted from the samples and then detected by MALDI-TOF-MS system to get the mass spectra. The mass spectra data was analyzed by the ClinproToolTM software to identify the different serum peptides, and then analyzed the clinical significance of peptides.

**Results:**

Among the 34 patients who received TKI treatment, there were none evaluated as complete response (CR), 11 patients evaluated as PR and 23 patients evaluated as stable disease (SD), with the PFS was 8.0 months (95%CI: 6.6-11.2); overall survival (OS) was 11.4 months (95%CI: 10.6-16.5). After detected the serum from three different points of time, the result showed that they were totally different; 87 different peptide peaks were identified after analysis self-paired serum between the time of best effect and baseline, which included one statistically different [*P* < 0.001, area under curve (AUC)≥0.9] peptide; 96 different peptide peaks were identified after analysis serum between the time of progression and baseline, which included 3 statistically different (*P* < 0.001, AUC≥0.9) peptides; 115 different peptide peaks were identified after analysis serum between the time of progression and best effect, which included 4 statistically different (*P* < 0.001, AUC≥0.9) peptides.

**Conclusion:**

Theserum peptides of NSCLC patients in the process of TKI treatment are dynamic and the different peptides may be associated with treatment effect and disease progression. However, the features and clinical significance of different peptides need to be validated in the future.

随着近年来肺癌的分子遗传学研究的进展，晚期非小细胞肺癌（non-small cell lung cancer, NSCLC）治疗步入了以表皮生长因子受体（epidermal growth factor receptor, *EGFR*）基因突变为代表的个体化分子靶向治疗新时代^[1]^。近年来*EGFR*基因检测方法不断推陈出新，循环肿瘤DNA（circulating tumor DNA, ctDNA）已作为组织标本检测的补充和替代^[2, 3]^。

然而，临床实践中我们发现即使*EGFR*突变阳性患者并非EGFR-酪氨酸激酶抑制剂（tyrosine kinase inhibitors, TKIs）治疗都敏感有效，考虑到除*EGFR*突变以外，下游信号变化等更多生物学改变对于TKI治疗效果存在影响。因此较单从基因突变角度分析而言，肿瘤蛋白组学研究蛋白表达可进一步精确预测疾病的发展变化。随着蛋白质谱技术平台的进步，自动化和高度平行检测分析血清蛋白质谱变化成为可能^[4-6]^。

为研究血液蛋白组学在NSCLC患者EGFR-TKI治疗过程中的改变，本研究使用MALDI-TOF质谱技术，动态分析治疗过程中不同时间节点的血清差异多肽并分析其临床意义。

## 1 材料和方法

### 1.1 样本来源

收集2011年10月-2013年5月在军事医学科学院附属医院肺部肿瘤科就诊经病理证实为晚期NSCLC患者接受EGFR-TKI治疗的血清。研究对象入选标准：①经组织学检测证实，病理类型为NSCLC；②患者分期为Ⅲb期或Ⅳ期[参照2009年第七版肺部肿瘤肿瘤-淋巴结-转移（tumor-node-metastasis, TNM）分期]；③肺内明确可测量病灶；④服用厄洛替尼、吉非替尼或埃克替尼，能够评价客观疗效且服用EGFR-TKI后生存时间不少于3个月；⑤对TKI的最佳总疗效均为疾病稳定（stable disease, SD）或部分缓解（partial response, PR），所有的患者随后均出现疾病进展（progressive disease, PD），参照2011年版实体瘤疗效评价标准（Response Evaluation Criteria in Solid Tumors, RECIST）；⑥患者可提供足够血清标本；⑦无其他原发恶性肿瘤；⑧所有患者均经临床检查排除重要脏器（心、肝、肾等）疾病；⑨所有患者采血均获得知情同意。

共有34例晚期NSCLC患者符合病例标准入组。本研究每例患者采血3次，分别为：①基线时：入组口服TKI治疗前采集血液标本；②最佳疗效时：临床评价PR或SD时所留取的血液标本；③疾病进展时：临床评价PD后所留取的血液标本。34例患者共收集102份血清，102份血清样本均成功完成质谱检测分析。患者基本情况见表 1。

**1:** 患者基本情况s Basic information of patientss

Characteristics	Data	Percent (%)
Age (yr)		
Median	57.5	/
Range	37-78	/
Gender		
Male	18	53
Female	16	47
ECOG PS		
0-1	28	82.4
2-3	6	17.6
Pathological type		
Adeno	28	82.4
Squamous	5	14.7
Else	1	2.9
Smoking history		
No	10	29.4
Current or former	24	70.6
EGFR status		
Mutant	14	41.2
Exon 19	8	23.5
Exon 21	6	17.7
Wild type	10	29.4
Unknown	10	29.4
TKI line of treatment		
1^st^	8	23.5
≥2^nd^	26	76.5
EGFR: epidermal growth factorreceptor; ECOG: Eastern Cooperative Oncology Group; PS: performance status; TKI: tyrosine kinase inhibitor.		

### 1.2 主要试剂和仪器

2%三氟乙酸、50%乙腈溶液、甲醇、混合标肽及铜离子螯合纳米磁珠（国家生物医学分析中心）、基质辅助激光解析离子化-时间飞行质谱仪（matrix-assisted laser desorption ionization time-of-flight mass spectrometry, MALDI-TOF-MS）（Ultraflex）、自动平衡离心机、纳米磁珠分离器（MBS），ClinProTools 2.1软件均为Bruker公司产品。

### 1.3 方法

#### 1.3.1 血清样品的采集

在患者签署知情同意后，晨起8:30空腹采集外周静脉全血3mL置于EDTA管常温下静置2 h；后于4 ℃、3, 500 r/min、离心10 min；取上清液（血清）100 μL至于-80 ℃冰箱保存待用。

#### 1.3.2 磁珠处理血清样品

① 吹打混匀磁珠，吸取5 μL磁珠及50 μL结合液置于样品管中；②冻融血清样本，吸取5 μL血清及20 μL结合液至样品管混匀，室温下于磁珠分离器内静置10 min，编号；③每管在磁珠分离器上移动4次，仔细用加样枪吸去悬浮液体→加入100 μL洗涤液→在磁珠分离器上移动4次，用加样枪弃去悬浮液；④每管加入100 μL洗涤液后在磁珠分离器上移动4次，直至悬浮液清澈用加样枪弃去，此步骤重复2次；⑤每管加入20 μL洗脱液，用加样枪反复吹打混匀，避免吸取磁珠，后静置于室温下20 min；⑥每支样品管于磁珠分离器上移动8次，室温下静置30 s直至悬浮液清澈；⑦将上清液移至新的样品管内重新编号，完成血清样本处理过程。

#### 1.3.3 点靶及质谱检测

① 为避免人为操作误差，MALDI-TOF仪器使用前用已知标准品进行误差校正；②清洗Anchorchip靶；③吸取基质（3 mg/mL HCCA、50%乙腈、2%三氟乙酸）与样品1:1混合，后置于室温待干燥后上机检测；④将样品靶放入MALDI-TOF仪器进行分析；⑤采用线性、正离子模式下打击样品，将峰谱累加至3, 000次，从而获得准确的由不同质荷（mass-to-charge ratio, m/z）的多肽峰构成的血清多肽指纹谱。

#### 1.3.4 统计学分析

应用ClinProTools 2.1软件对MALDI-TOF质谱仪采集数据进行处理：首先进行Top H at Baseline基线处理和Savitsky Golay Smoothing平滑处理，并过滤掉信噪比 < 4的峰，将数据校正和归一化（TIC总离子流）。应用峰面积作为选用蛋白峰量化指标，得到处理后图谱。结果由随机软件ClinPro Tools（Version: 2.1）采集信号并进行数据分析。血清蛋白峰分析统计方法为配对样本*t*检验，规定曲线下面积（area under curve, AUC）≥0.9，*P* < 0.001的多肽峰有统计学差异。

## 2 结果

### 2.1 所有入组患者的一般临床特征

共入组34例患者，所有患者具体情况见表 1。

### 2.2 疗效评价

34例接受EGFR-TKI治疗的NSCLC患者按RECIST 1.1标准，最佳疗效为CR共0例，PR共11例，SD共23例。从开始口服TKI治疗起计算，PFS为8.0个月（95%CI: 6.6-11.2），OS为11.4个月（95%CI: 10.6-16.5）。以OS为主要研究终点，随访至2015年12月，共有31例患者死亡，3例带瘤生存，生存曲线见图 1。

**1:**
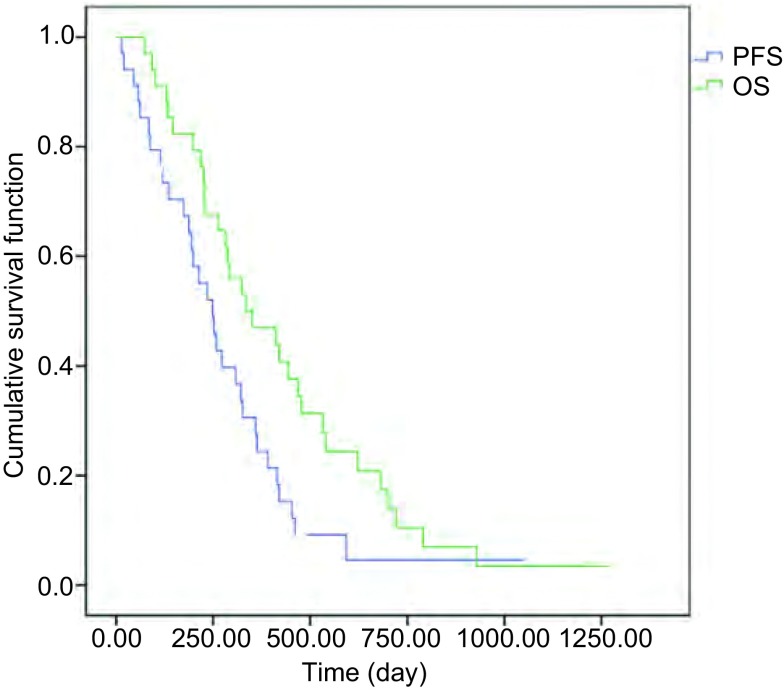
34例患者生存曲线 *Kaplan*-*Meier* curves of survival of all patients. PFS: progression-free survival; OS: overall survival.

### 2.3 血清多肽结果

#### 2.3.1 血清多肽指纹图谱的获得

应用ClinProTools 2.1软件对MALDI-TOF质谱仪采集数据进行处理，分别得出34例患者整体基线时、最佳疗效时及疾病进展时多肽指纹图谱，结果见图 2。初步观察，发现3个时间点指纹图谱均不相同。为进一步明确其间差异多肽峰，使用统计学软件对结果分别进行差异鉴定。

**2:**
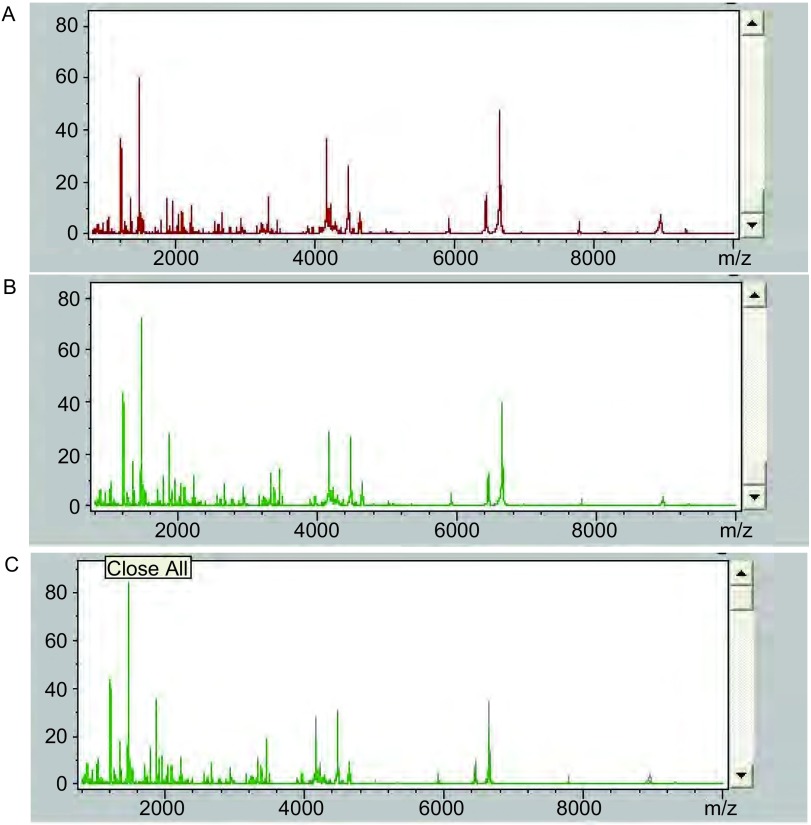
血清多肽指纹图谱。A：基线时；B：最佳疗效时；C：疾病进展时。 Serum peptide profiles of 3 point of time. A: Baseline; B: Best effect; C: Progression of disease.

#### 2.3.2 基线时与最佳疗效时血清自身配对差异多肽结果

最佳疗效时与基线时质谱数据经统计学软件分析后共鉴定出差异多肽峰87个，筛选出两组间有统计学差异（*P* < 0.001, AUC≥0.9）的多肽峰1个，差异表达多肽是1, 897.16（m/z）。该多肽由基线时表达水平251.39±277.83，下调为最佳疗效时的36.19±32.01。

#### 2.3.3 基线时与疾病进展时血清自身配对差异多肽结果

疾病进展时与基线时经统计学软件分析后共鉴定出差异多肽峰96个，筛选出两组间有统计学差异（*P* < 0.001, AUC≥0.9）的多肽峰3个，具体结果见表 2。差异表达蛋白分别是7, 476.88、6, 192.62及7, 571.91（m/z）。与基线时相比，最佳疗效时m/z为7, 476.88及7, 571.91的多肽表达下调，m/z为6, 192.62的多肽表达上调。

**2:** 基线时与疾病进展时差异多肽质荷比及表达变化 m/z and expression change between the time of baseline and progression of disease

m/z	Baseline	Progression of disease	Expression levels of the progression of disease compared to the baseline
7, 476.88	2.39±1.41	1.04±0.51	↓
6, 192.62	26.4±19.63	57.84±29.73	↑
7, 571.91	3.22±1.99	1.56±0.83	↓
*P* < 0.001; AUC≥0.9. AUC: area under curve; m/z: mass-to-charge ratio.			

#### 2.3.4 最佳疗效时与疾病进展组血清自身配对差异多肽结果

最佳疗效时与疾病进展时经统计学软件分析后共鉴定出差异多肽峰115个，筛选出两组间有统计学差异（*P* < 0.001, AUC≥0.9）的多肽峰4个，具体结果见表 3。差异表达蛋白分别是8, 946.26、7, 933.99、4, 210.20及5, 338.48（m/z）。与最佳疗效时相比，疾病进展时4个多肽均下调。

**3:** 最佳疗效时与疾病进展时差异多肽质荷比及表达变化 m/z and expression change between the time of best effect and progression of disease

m/z	Best effect	Progression of disease	Expression levels of the progression of disease compared to the best effect
8, 946.26	2.54±2.36	0.77±0.36	↓
7, 933.99	3.08±2.86	0.95±0.79	↓
4, 210.20	9.86±9.26	3.37±1.68	↓
5, 338.48	119.19±77.9	62.73±35.14	↓
*P* < 0.001; AUC≥0.9.			

#### 2.3.5 相关性分析

通过质谱软件分析，最佳疗效时与基线时自身配对得出1个差异多肽峰（1, 897.16），疾病进展时与基线时自身配对得出3个差异多肽峰（7, 476.88、6, 192.62及7, 571.91），最佳疗效时与疾病进展时自身配对得出4个差异多肽峰（8, 946.26、7, 933.99、4, 210.20及5, 338.48）。

将两两比较共检测到的8个差异多肽峰根据*P*值从低到高的顺序进行排列，结合临床预后参数PFS和OS，并与*EGFR*突变情况进行相关分析。发现最佳疗效时与基线时自身配对得出的m/z为1, 897.16差异多肽峰与PFS有相关性。疾病进展时与基线时自身配对得出3个差异多肽峰中，2个多肽峰（6, 192.62及7, 571.91）与PFS有相关性，m/z为7, 476.88的多肽峰与OS有统计学意义的相关。最佳疗效时与疾病进展时自身配对得出4个差异多肽峰中，7, 933.99与OS有统计学意义的相关，3个多肽峰（8, 946.26、4, 210.20及5, 338.48）与PFS及OS均无相关性。在24例行基因突变检测的患者中，14例为*EGFR*基因突变型患者10例为野生型患者。将*EGFR*突变检测结果与8个差异多肽峰进行相关分析，得出二者之间没有统计学差异。

## 3 讨论

对于*EGFR*基因检测，目前推荐肿瘤组织标本检测的基础上，血液（血浆）标本检测ctDNA成为补充手段。与基因检测相比蛋白质组学包含着基因组学表达、执行以外的生物学信息^[7-9]^，可进一步有助于我们深入了解和分析影响肺癌靶向治疗敏感性的复杂因素。质谱技术可以对标本中所有的蛋白或多肽进行检测和质量测定，能够找到其他检测方法可能发现不到的特定分子量的物质^[10-12]^。

近年来，使用磁珠辅助MALDI-TOF-MS分析NSCLC患者EGFR-TKI治疗前血清发现，通过该方法建立的分类模型，可用于预测TKI治疗效果，从而辅助指导NSCLC患者个体化治疗。Taguchi等^[13]^利用MALDI-TOF检测139例接受TKI治疗前的NSCLC患者血清。通过该方法建立的VeriStrat分类模型可以有效地将96例服用TKI的患者分为“好”（good，中位生存期为306天）和“差”（poor，中位生存期为107天）两组，且这两组EGFR-TKI治疗后的TTP也有明显差异，分别为3.2个月和1.9个月（HR=0.53, 95%CI: 0.33-0.85, *Log*-*rank*: *P*=0.007）。该模型在多中心证实有良好的重复性，可通过检测TKI治疗前患者血清，鉴定TKI获益人群。Wu等^[14]^的实验对象为68例使用TKI进行二线或三线治疗的NSCLC患者TKI治疗前血请。首先使用MALDI-TOF检测训练组血清，建立由3个多肽组成的分类模型。使用该模型盲法将44例测试组血清分为“好”组与“差”组的准确性为93%。“好”组的OS与PFS明显高于“差”组。该实验进一步证实了使用蛋白分级算法预测NSCLC患者TKI治疗效果的可行性。同时既往我们实验组杨琳等^[15]^通过检测训练组100例晚期NSCLC患者TKI治疗前血清，得出*EGFR*基因TKI敏感型突变与野生型*EGFR*基因差异多肽，建立分级算法。该算法在后续123例确认组中证实有良好的敏感性和特异性。

然而，对于EGFR-TKI治疗过程中及疾病进展后的血清蛋白组学发生了怎样的变化缺乏系统研究。既往杨学宁等^[16]^收集9例晚期NSCLC患者接受吉非替尼（gefitinib）治疗前后自身配对的血清样本20份。使用MALDI-TOF血清蛋白质谱检测，发现有7个血清蛋白质表达水平在耐药前后的血清中有统计学差异，且其中6个差异多肽与TTP相关，但与OS均不相关。但遗憾的是，该实验样本例数较少，需要在扩大样本量的基础上对结果加以验证。

为了明确分析EGFR-TKI治疗全程血清蛋白组学变化，我们选取34例晚期NSCLC患者TKI治疗前、最佳疗效时及疾病进展后的自身配对血清样本102份进行分析。入组的34例患者从开始口服TKI治疗起计算，中位PFS为8.0个月，中位总生存期OS为11.4个月，其中TKI治疗时机为一线的8例患者PFS为9.1个月，OS为11.3个月，二线及之后的26例患者PFS为7.8个月，OS为11.1个月。结果均与大部分临床试验数据接近。

对患者得出基线时、最佳疗效时及疾病进展时三个时间点多肽指纹图谱通过肉眼观察，可大致发现三个指纹图谱均不相同。进一步进行两两比较，得出有统计学意义的多肽峰：①最佳疗效时与基线时自身配对得出1个差异多肽峰（1, 897.16）；②疾病进展时与基线时自身配对得出3个差异多肽峰（7, 476.88、6, 192.62及7, 571.91）；③最佳疗效时与疾病进展时自身配对得出4个差异多肽峰（8, 946.26、7, 933.99、4, 210.20及5, 338.48）。

将两两比较时共检测到的8个差异多肽峰根据*P*值从低到高的顺序进行排列，结合临床预后参数PFS和OS，并与*EGFR*突变情况进行相关分析。发现最佳疗效时与基线时自身配对得出的m/z为1, 897.16差异多肽峰与PFS有相关性，由此推测在患者治疗获益时血清可检测到一过性改变，且其与疗效预测相关。疾病进展时与基线时自身配对得出3个差异多肽峰中，2个多肽峰与PFS有相关性，m/z为7, 476.88的多肽峰与OS有统计学意义的相关。最佳疗效时与疾病进展时自身配对得出4个差异多肽峰中，7, 933.99与OS有统计学意义的相关，3个多肽峰与PFS及OS均无相关性。与杨学宁的结论不同在于，我们分析出的差异多肽与OS存在相关性。原因可能在于此次样本例数的扩大，避免了因为样本例数不足而未能与OS产生相关的可能。另外，这些有差异的多肽其性质是什么、特点和功能如何，还需我们今后的多肽功能分析工作中得以验证。

该研究以MALDI-TOF质谱技术为支撑，通过对肺癌患者血液中多肽组的变化，单纯检测*EGFR*基因突变相比无疑包含着*EGFR*突变下游信号变化等更多生物学信息，因此较单从基因突变角度分析更全面。

然而，因本研究仅为单中心、小样本的探索性研究，且34例患者接受EGFR-TKI治疗时机有所不同，虽然通过自身配对减少个体代谢差异等生物学差异对结果的解释偏倚，但仍不可避免的给本研究带来了混杂因素。所以未来还需要扩大样本量，进行设计严谨的前瞻性研究。此外，为了明确差异多肽的具体性质，应对差异多肽进行分离纯化鉴定以进一步研究其结构功能，探讨其对于TKI治疗的指导意义。
